# Obesity, diabetes and OSAS induce of sleep disorders: Exercise as therapy

**DOI:** 10.1186/1476-511X-10-148

**Published:** 2011-08-23

**Authors:** Eduardo S Alves, Fabio S Lira, Ronaldo VT Santos, Sergio Tufik, Marco T de Mello

**Affiliations:** 1 Departamento de Psicobiologia, Universidade Federal de São Paulo, Brazil; 2 Centro de Estudos em Psicobiologia e Exercício - CEPE, São Paulo, Brazil; 3 Departamento de Biociências, Universidade Federal de São Paulo, Campus Baixada Santista, Brazil

## Abstract

Sleep is an integral part of good health. Sleep disorders and variations in sleep habits are associated with a low-grade inflammatory status, which may be either a cause or consequence of other conditions, including obesity, diabetes and cardiovascular disease. Several strategies are available to counteract these conditions including continuous positive airway pressure (CPAP), pharmacological and nutritional interventions, and even surgery. At present, our group is investigating the effect of chronic endurance exercise on sleep alterations.

## Hypothesis

Sleep deprivation is a common phenomenon in today's society. Over the last 50 years, daily sleep duration in adolescents and adults decreased by 1.5-2 hours, and more than 30% of Americans between the ages of 30 to 64 years old report less than 6 hours of sleep per night [1]. Moreover, the quality of sleep decreases with age [2]. In addition, the prevalence of obstructive sleep apnea syndrome (OSAS), obesity, and cardiovascular and metabolic disease are increasing [3]. This is relevant because epidemiological studies report a positive correlation between decreased sleep time and increases in body mass index and diabetes prevalence [4,5].

OSAS is a common disorder characterized by repetitive episodes of partial or complete obstruction of the upper airway during sleep and increased respiratory effort. This syndrome can lead to the development of obesity and diabetes [6,7].

Different strategies are utilized to counteract OSAS, obesity and diabetes [8]. CPAP treatment exerts a beneficial effect on glucose metabolism and insulin resistance in people with OSAS [9]. However, when CPAP usage ceases the positive effects are abolished. Others therapies, such as drugs to aid weight loss and normalize insulin are often used to treat obesity and diabetes, respectively. Although these approaches target individual problems, we hypothesize that chronic endurance exercise may be an effective treatment for all three conditions.

Our group has previously demonstrated the effects of acute and chronic exercise on sleep in both humans and rats [[10-12], *unpublished*]. Exercise training improves outcomes, including total adipose tissue and diabetes, and may ameliorate OSAS.

**Figure 1:**
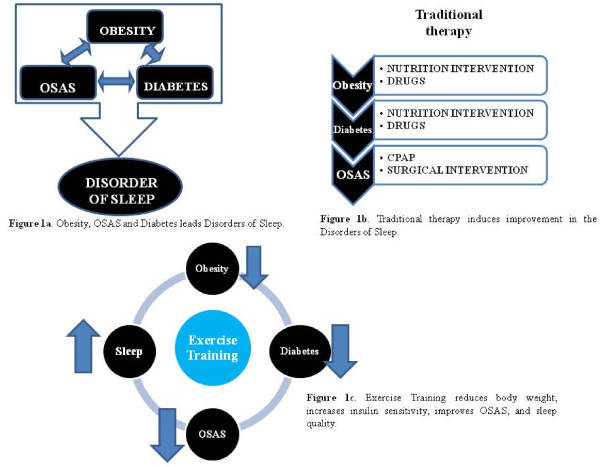
**Exercise training compared with conventional therapies for counteracting OSAS, obesity and diabetes involved in the development of sleep disorders**.

Confirming our hypothesis, Figure 1 shows that compared with conventional therapies, exercise training is a more effective strategy for counteracting OSAS, obesity and diabetes involved in the development of sleep disorders. However, additional studies are needed to elucidate the mechanism(s) of how exercise training improves sleep quality.
